# Molecular Characteristics of Fat Body Protein 1 in the Oriental Fruit Fly, *Bactrocera dorsalis*

**DOI:** 10.3390/insects12040319

**Published:** 2021-04-02

**Authors:** Yao-Chih Yu, Hsuan Lu, Yi-Cheng Chiang, Cheng-Lung Tsai, Yu-Han Zuo, Mei-Er Chen

**Affiliations:** 1 Department of Entomology, National Chung-Hsing University, Taichung City 40227, Taiwan; crazy_request@hotmail.com (Y.-C.Y.); bonny851026@gmail.com (H.L.); yichengchiang88@gmail.com (Y.-C.C.); lillian-house@yahoo.com.tw (Y.-H.Z.); 2 Ft. Lauderdale Research & Education Center, Department of Entomology and Nematology, University of Florida, Ft. Lauderdale, FL 33314, USA; cltsai.lucanid@gmail.com

**Keywords:** *Bactrocera dorsalis*, fat body protein 1, transcriptional expression, eclosion

## Abstract

**Simple Summary:**

*Bactrocera dorsalis* fat body protein 1 (*Bdfbp1*) cDNA was cloned. The deduced amino acid sequence contains three motifs: hemocyanin N, high molecular weight glutenin (gultenin hmw), and hemocyanin C from N to C termini. The glutenin hmw allows Bdfbp1 to fold into a compact form for storage. *Bdfbp1* was highly expressed in the late third instar larvae and day 0 pupae. This suggests that Bdfbp1 is stored during larval stages as a storage protein for construction of adult tissues during pupal stages, and may be associated with adult eclosion.

**Abstract:**

*Bactrocera dorsails* fat body protein 1 (*Bdfbp1*) cDNA was cloned (GenBank accession no. MT514270), and the complete 3,749-bp cDNA encoded a 1,152-amino acid protein. The phylogenetic relationship of dipteran *fbp1s* was analyzed. The sequence XP_028900815 from the insect genome project for *Zeugodacus cucurbitae* (LOC105219342) was proposed that two *fbp1* genes were present in the sequence. The developmental transcriptional expression profiles were determined. In the larval stages, *Bdfbp1* mRNA had significantly higher expression in the late third instar larvae compared with first, second, and early third instar larvae. In the pupal stages, the highest expression of *Bdfbp1* mRNA was found in the newly pupated pupae and then decreased with age. In the fat body of female adults, *Bdfbp1* was highly expressed in newly emerged samples and decreased rapidly over the following three days. In the fat body of male adults, *Bdfbp1* was highly expressed in newly eclosed samples. RNAi treatment decreased the expression level of *Bdfbp1* without statistical difference. However, RNAi treatment significantly decreased the rate of eclosion. These results suggest that Bdfbp1 may function as a storage protein and be associated with adult eclosion.

## 1. Introduction

The fat body, an essential tissue for insect metabolism, provides energy and synthesizes proteins for insect development and reproduction. As the developmental stages change, the function of the fat body changes accordingly. At the pupal stage, insects require large amounts of energy and materials for adult development. Because pupae do not ingest any food, all substances for adult development are stored during the larval stages, especially the late larval stage.

The oriental fruit fly, *Bactrocera dorsalis*, is one of the most disastrous pests, leading to damages of about 450 plant species throughout the world [1]. Their high fecundity makes them preeminent, with 1000 eggs laid over the entire life-span of females [2]. Females deposit their eggs into fruit where the larvae hatch and feed. Until the late third instar, larvae drill out the fruit and enter the soil to pupate. Pesticides are most frequently employed to control *B. dorsalis*. The tendency to develop resistance to different types of insecticides has been reported [3,4,5]. It is necessary to develop new strategies to control *B. dorsalis*.

There are two forms of fat body in the *B. dorsalis* adult abdomen: the white, nodule-like larval-form fat body and the thin, sheet-like adult-form fat body [6]. A comparison of gene expression between the larval- and adult-form fat body in *B. dorsalis* adults reveals that the arylphorin receptor is expressed more abundantly in the larval form [6]. The nomenclature of the arylphorin receptor (accession no. JZ476873) was changed to fat body protein 1 (*fbp1*) after the release of *B. dorsalis* genome annotation.

The *fbp1* gene of *Drosophila melanogaster* has long served as a model for the study of 20-hydroxyecdysone regulation. The transcriptional expression of *fbp1* is the tissue specific in the fat body, and temporally specific at the end of the third instar [7,8]. A ligand binding assay and Western blot analyses demonstrated that the product of *fbp1* of *D. melanogaster* is a larval serum protein (LSP) receptor [9]. LSPs are storage proteins generally referred to as hexamerins, which are involved in pupation and metamorphosis [10,11].

In *B. dorsalis*, the storage protein, hexamerin gene *BdAr* is expressed in third instar larvae, pupae, and adults, with especially high expression levels in late third instar larvae and day 0 adults [12]. The form of fat body is larval form in third instar larvae and day 0 adults. Because *B. dorsalis fbp1* (*Bdfbp1*) is highly expressed in larval-form fat body as *BdAr* is [6], this makes us to hypothesize that fbp1 functions as a storage protein. The developmental transcription profiles of *Bdfbp1* were determined through real-time quantitative polymerase chain reaction, and the function of Bdfbp1 related to adult eclosion was explored through RNA interference.

## 2. Materials and Methods

### 2.1. Insects

Colonies of the oriental fruit fly, *B. dorsalis*, were maintained as described elsewhere [12]. Larvae were fed on an artificial diet containing 1800 mL of water, 20 mL of HCl, 5 g of benzoic acid, 240 g of sugar, 140 g of yeast extract, and 480 g of wheat bran [13].

### 2.2. cDNA Cloning of fbp1 through Rapid Amplification of cDNA Ends and Sequence Analyses

A 641-bp cDNA fragment encoding partial *Bdfbp1* was obtained from the day 0 (D0) fat body cDNA library [6]. The 5′ and 3′ end of the *Bdfbp1* cDNA fragments were produced using the GeneRacer^TM^ Kit (Thermal, Carlsbad, CA, USA). The total RNA was extracted from the D0 female fat body using TRIZOL^®^ Reagent (Thermal, Carlsbad, CA, USA) according to the manufacturer’s instructions. *Bdfbp1*-specific primers were designed based on the sequence of the 641-bp fragment. The PCR parameters were as follows: 94 °C for 2 min; 35 cycles of 94 °C for 30 s, 56–62 °C (based on the Tm of the primers, Table 1) for 30 s, and 72 °C for 1–3 min (based on the expected size of the fragment); and 72 °C for 10 min. The PCR products were purified, cloned, and sequenced as described elsewhere [12].

To prevent errors during assembly, the entire open reading frame in one fragment was cloned using two specific primers designed in the 5′ and 3′ untranslated regions. The sequences of sense and antisense primers were: *Bdfbp1*-full-01 5′-GACATTGATTGGTAGAGTCCAGCGGATAC-3′ and *Bdfbp1*-full-02 5′-GCTTCGGCTAATGCAACTAGCAGTGAG-3′, respectively. The PCR parameters were as follows: 94 °C for 2 min; 40 cycles of 94 °C for 30 s, 60 °C for 30 s, and 72 °C for 4 min; and 72 °C for 10 min. 

The signal peptide was predicted using the SignalP 4.1 Server [14] (http://www.cbs.dtu.dk/services/SignalP4.1/). The Conserved Domains Database (CDD, http://www.ncbi.nlm.nih.gov/Structure/cdd/wrpsb.cgi) (accessed on 25 May 2020) on the NCBI website was used to identify the conserved motifs [15]. Lalign program [16] was applied to align amino acid sequences of MT514270 and XP_029405180.

### 2.3. Identification of the fbp1 from the Melon Fly, Zeugodacus Cucurbitae

The blastn and blastp search algorithms in NCBI searches indicated that the sequence cloned in this study had high identity to the *B. dorsalis* fat body protein 1 (accession numbers: XM_029549320 and XP_029405180, respectively). The blast searches also revealed one uncharacterized gene product from the genome of the melon fly, *Zeugodacus cucurbitae*, (GeneID: LOC105219342; protein accession number: XP_028900815). A protein of 2380 amino acids was identified from XP_028900815. Blast results revealed that the amino acid sequence 1–1225 of XP_028900815 was highly similar to that of the *Bdfbp1* cloned in this study. However, a melon fly *fbp1* (accession number: JAD03259) was found in a survey of the GenBank protein database and its sequence was same as the amino acid sequence 130–2380 of XP_028900815 from LOC105219342.

### 2.4. Phylogenetic Analysis

Amino acid sequences of 11 *fbp1s* from the blast results and GenBank were used for the phylogenetic analysis. Sequences were aligned using the MUSCLE program (http://www.ebi.ac.uk/Tools/msa/muscle/) (accessed on 20 July 2020). Phylogenetic relationships were analyzed using neighbor-joining (NJ). The Poisson model was used as substitution model to construct an NJ tree using MEGA 7.0 software [17]. Bootstrapping analyses of 1000 replications were applied.

### 2.5. Real-Time Quantitative PCR (qPCR)

Developmental regulation of *Bdfbp1* transcription was performed in larvae, pupae, and adult fat body tissues through qPCR. The sample collection of different developmental stages and qPCR were conducted [12]. Briefly, larvae were collected from the first, second, early third, and late third instars. Pupae were collected separately from days 0 to 8 after pupation. Fat bodies from 0- to 10-day-old females and males were separately isolated. Glyceraldehyde-3-phosphate dehydrogenase (gapdh) was used as the reference gene for adult samples [18]. *18S* ribosomal RNA genes were used as the reference genes for larval and pupal samples. The sequences of specific primers used in the qPCR are listed in Table 2. The PCR parameters were as follows: 50 °C for 2 min; 95 °C for 8.5 min; and 40 cycles of 95 °C for 15 s and 60–65 °C (based on the Tm of the primers, Table 2) for 1 min. A melting curve analysis was carried out for each test to determine the specificity of the amplification. Three independent biological replicates of each developmental stage were performed. The expression data were collected via the Bio-Rad iQ5 2.0 Standard Edition Optical System Software V2.0. The expression data were applied for quantification data analyses. The statistical analyses, ANOVA followed by a Tukey multiple comparison were performed using Prism™ 5.0 (GraphPad Software, San Diego, CA, USA). *P* < 0.05 was considered statistically significant.

### 2.6. RNA Interference

Synthesis of double-stranded *Bdfbp1* (ds*Bdfbp1*) RNA was conducted using MEGAscript^®^ RNAi Kit (Thermal) following the manufacturer’s instructions. A plasmid containing the full-length cDNA of *Bdfbp1* was used as the template. The two pairs of primers used to synthesize the dsRNA are listed in Table 3. The dsRNA products were electrophoresed on 1% agarose gel to confirm the size and integrity. For the feeding treatment, 40–60 eggs were placed into a Petri dish (15 cm in diameter) containing 55 g of larval artificial diet. In total, 50 μg of two fragments of dsRNA (25 μg of each) in 10 mL of water was added to the Petri dish on day 3 after egg collection. At this time, the larvae were in the early third instar. Ten mL of water was added to the Petri dish for the controls. After five days of continuous feeding (250 μg of dsRNAs or water), late third instar larvae started to jump out the artificial diet for pupation. Ten late third instar larvae were collected from each treatment and control groups for RNA extraction, and one μg of RNA was reverse-transcribed as cDNA for qPCR. The others were left for pupation. Pupae were then moved to observe the rate of eclosion. The rate of eclosion was analyzed using Student’s *t* test. *P* < 0.05 was considered statistically significant.

## 3. Results

### 3.1. cDNA Cloning and Sequence Analyses

**Figure 1:**
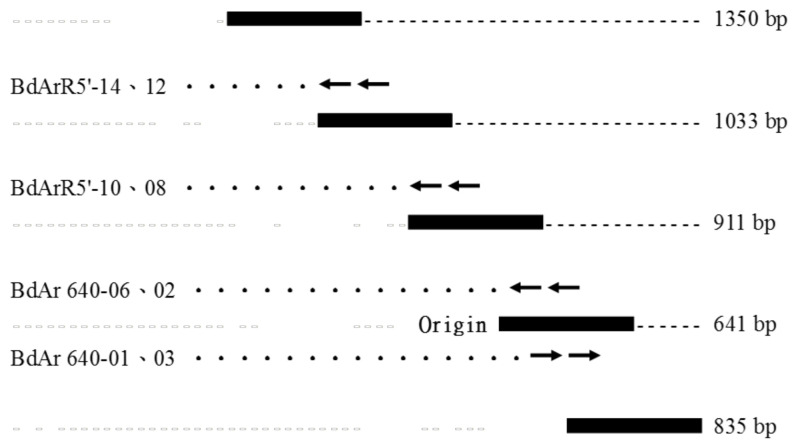
Assembly of the fragments of the *fbp1* cDNA cloning in *B. dorsalis*.

**Figure 2:**
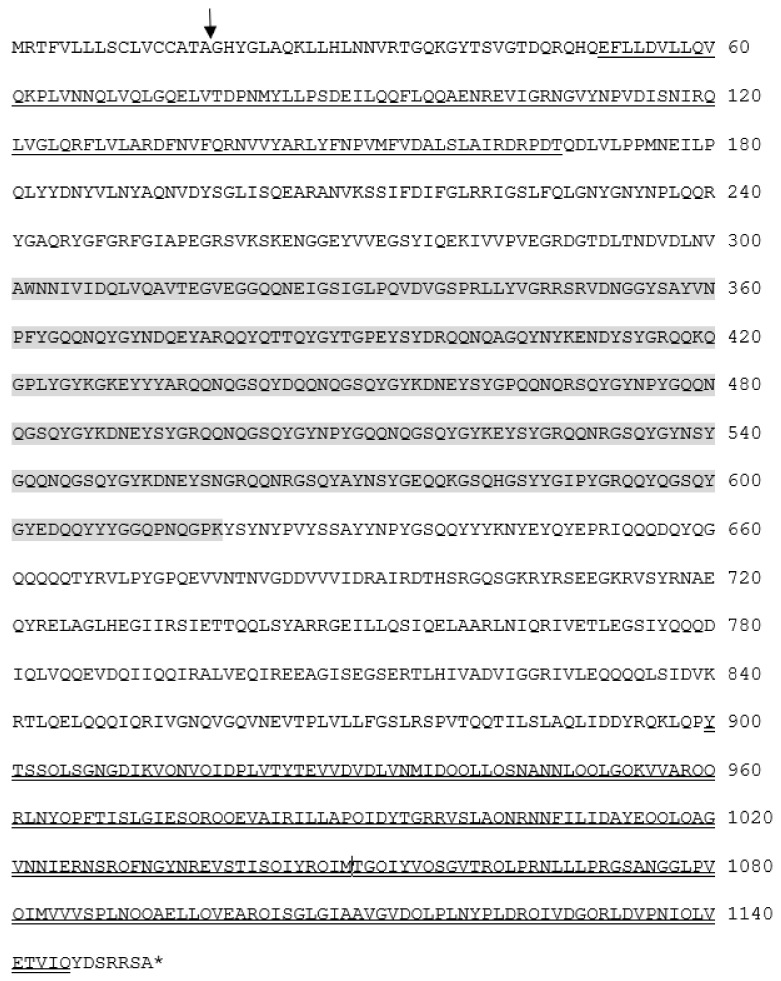
Deduced amino acid sequences of the fat body protein 1 cDNA from *B. dorsalis* (GenBank accession number MT514270). The cleavage site of the putative signal peptide sequence is indicated by a vertical arrow. The hemocyanin N domain is underlined, the hemocyanin C domain is indicated with double underlines, and the high molecular weight glutenin subunit is marked in gray.

The five cDNA fragments encompassing the entire coding region of the *Bdfbp1* cDNA were cloned, sequenced, and analyzed (Figure 1). The assembled 3749-bp cDNA sequence revealed an open reading frame of 3459 bp encoding a protein of 1152 amino acid residues (GenBank accession no. MT514270). Signal 4.1 predicted that the first 17 amino acids at the N-terminus produced a signal peptide and no transmembrane domain was predicted. The CDD search for the *Bdfbp1* revealed that the N (amino acid sequence 50–167) and C (amino acid sequence 899–1145) termini belonged to hemocyanins N and C, respectively. The conserved motif between hemocyanins N and C was a high-molecular-weight glutenin subunit (glutenin hmw, amino acid sequence 361–618) (Figure 2).

**Figure 3:**
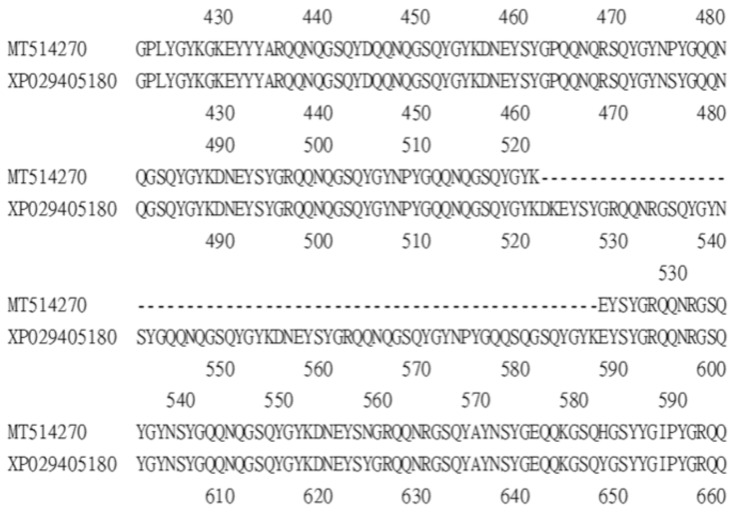
Alignment between *Bdfbp1* MT514207 (amino acid sequence 421–594) and *Bdfpb1* XP029405180 (amino acid sequence 421–660) shows that MT514207 is 66 amino acids shorter than XP029405180.

**Figure 4:**
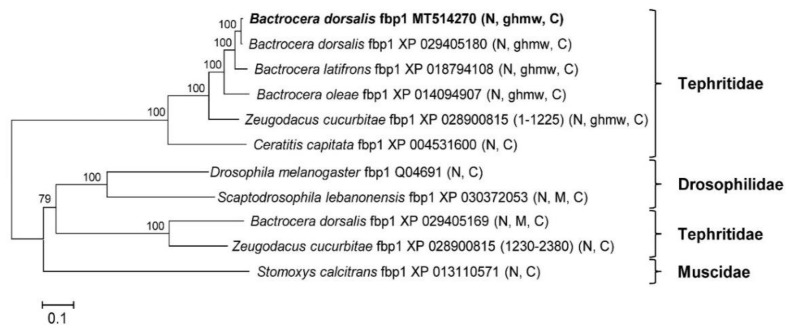
The phylogenetic tree of 11 versions of insect fat body protein 1 (*fbp1*) was constructed by neighbor joining. Numbers at nodes are based on bootstrapping statistical support. Scale bar = 0.1 substitution/site. Conserved domains in parentheses are preceded by the scientific name, and the accession number. N: hemocyanin N; M: hemocyanin M; C: hemocyanin C; ghmw: high molecular weight glutenin subunit.

A survey of the GenBank protein database identified two *Bdfbp1s* (accession numbers XP_029405180 and XP_029405169). The identity between the protein cloned in this study, MT514270, and the protein from GenBank, XP_029405180, was 94%. The alignment between MT514270 and XP029405180 indicated that MT514270 was almost identical to XP_029405180, except that MT514270 was 66 amino acids shorter than XP_029405180 (amino acid sequence 522–587) (Figure 3). These 66 amino acids belong to the glutenin hmw motif. The identity between MT514270 and XP029405169 was 23.7%. No glutenin hmw motif was found in XP_029405169 (Figure 4). 

Eleven amino acid sequences from the GenBank protein database were applied to the phylogenetic analysis, including the *Z*. *cucurbitae* sequence XP_028900815, which was separated into two sequences (amino acid sequences 1–1225 and 1230–2380). We found that *Z*. *cucurbitae* JAD03259 was 100% identical to the sequence 1230–2380 of XP_028900815 from the genome of *Z. cucurbitae*. A phylogenetic analysis revealed that XP_028900815 contained two genes (Figure 4). The results also showed that all *fbp1s* were grouped into two clades. One clade contained only Tephritidae and the other contained Drosophilidae, Tephritidae, and Muscidae (Figure 4). The conserved motifs of the distinct Tephritidae clade were hemocyanin N, glutenin hmw, and hemocyanin C from the N to C termini. *Ceratitis capitata fbp1* was an exception; it contained only hemocyanins N and C. The other clade contained two types of conserved motifs: hemocyanins N and C; and hemocyanins N, M, and C from the N to C termini (Figure 4). The difference between the two clades is that the glutenin hmw.

### 3.2. Expression Profiles of Bdfbp1

**Figure 5:**
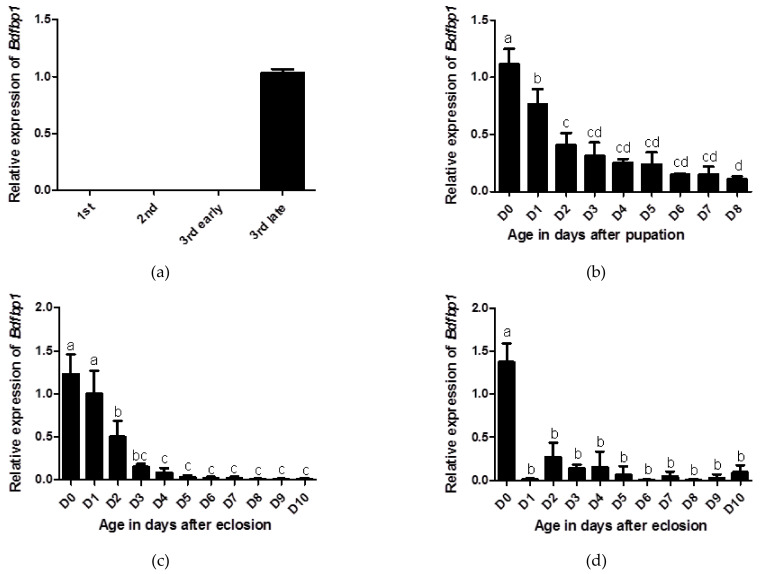
Developmental profiles of *Bdfbp1* transcription were determined through qPCR. (**a**) larval stage: first, second, early third, and late third instar; (**b**) pupal stage: 0–8 days after pupation; (**c**) adult female fat body, and (**d**) adult male fat body: 0–10 days after eclosion. Statistical analyses using ANOVA followed by a Tukey multiple comparison test were performed to identify differences between individual means. The same letters indicate no significant difference (*p* > 0.05). Standard deviation of three independent biological replicates is indicated by error bars in (**a**–**d**).

The developmental transcriptional expression profiles of *Bdfbp1* were determined through qPCR. In the larval stages, *Bdfbp1* transcripts were expressed at significantly higher levels in the late third instar compared with the first, second, and early third instar (Figure 5a). The expression level for day 0 pupae was the highest, followed by day 1 pupae, and these differences in expression levels were statistically significant (Figure 5b). After eclosion, *Bdfbp1* transcripts were detected in the female fat body from day 0 to day 10. The expression level was highest on days 0 and 1 with significantly higher expression levels than on day 2 to day 10 (Figure 5c). Although the expression levels of *Bdfbp1* were high in the day 0- and day 1-female fat body, they were still lower than the expression levels in the day 7 and day 8 pupae (data not shown). In the male fat body, *Bdfbp1* transcripts were also detected from day 0 to day 10. However, *Bdfbp1* had significantly higher expression levels on day 0 (Figure 5d).

### 3.3. Functional Studies of Bdfbp1

**Figure 6:**
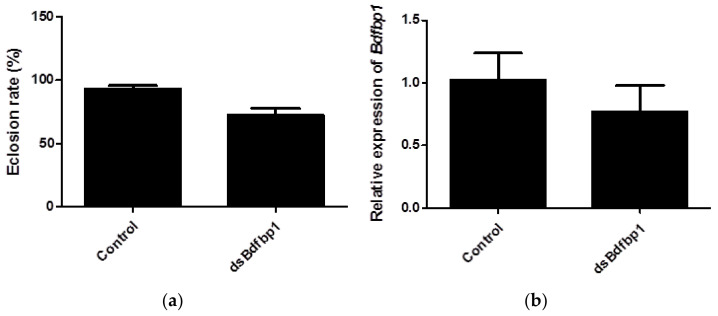
(**a**) The eclosion rate of *B. dorsalis* after RNAi treatment of larvae; (**b**) *Bdfbp1* relative transcriptional expression in *B. dorsalis* late third instar larvae after RNAi treatment. Statistical analyses were performed using Student’s t test. The asterisk indicates significant difference (*p* < 0.05). The standard deviation of each triplicate is shown by error bars in (**a**,**b**).

In this study, RNAi was applied to investigate the function of Bdfbp1 as a storage protein for metamorphosis. After 5 continuous days of larval feeding of ds*Bdfbp1* RNA or water, both treatment and control larvae were able to pupate normally. However, the eclosion rate of pupae was 72.2% in the treatment group compared with 93.2% in the control group. The difference was statistically significant (*p* = 0.0041; Figure 6a). The expression of *Bdfbp1* was 25% lower than that of the control after ds*Bdfbp1* RNA application, although this difference was not statistically significant (*p* = 0.2240; Figure 6b).

## 4. Discussion

The present studies were undertaken to clone *fbp1* cDNA from *B. dorsalis* as a first step in investigating the transcriptional expression profiles and the function of Bdfbp1. The deduced amino acid sequence of *Bdfbp1* indicated that it secretes into the hemolymph because there was a signal peptide predicted at the N terminus and no transmembrane domain was predicted (Figure 2). Bdfbp1 contained the hemocyanin N, glutenin hmw, and hemocyanin C domains from N to C termini. Glutenin hmw has an extensive central elastomeric domain flanked by two nonelastic domains. The central elastomeric domain is characterized by three repeated motifs, namely PGQGQQ, GYYPTSPQQ, and GQQ [19]. These motifs confer elasticity to the fbp1, allowing it to fold into a more compact storage protein.

In the GenBank protein database, all *fbp1s* are from insects and most of them are from Brachycera, Diptera. The nomenclature for *fbp1* is rather disordered. We found that two *fbp1* genes were included in *Z. cucurbitae* sequence XP_028900815. One contains glutenin hmw motif, and the other does not (Figure 4). The one without the glutenin hmw motif is also assigned as another accession number JAD03259. There are two *Bdfbp1s* found in the GenBank, XP_029405180 and XP_029405169. Based on the phylogenetic analysis, these two *Bdfbp1s* are belonged to different clades (Figure 4). *Bdfbp1* cloned in this study, MT514270, is more related to or same as XP_029405180 than it is to XP_029405169 (Figure 4). The aforementioned results suggest that the phylogeny, nomenclature, and evolution of these molecules are complex. It would be difficult to correctly summarize the characteristics of *fbp1* without further studies being conducted. 

*Bdfbp1* transcriptional expression is developmentally regulated (Figure 5). The transcriptional expression of *Bdfbp1* in the first, second, and early third instar was difficult to detect but the expression level was the highest in the late third instar. This expression profile was similar to that of *Drosophila* [7,8]. During the pupal stages, *Bdfbp1* transcripts were expressed constantly from day 0 to day 8 after pupation, showing a trend to decrease with age. To date, no studies have reported transcriptional expression of *fbp1* in the pupal stages. On the basis of expression profiles of larvae and pupae, it has been suggested that Bdfbp1 is stored during larval stages as a storage protein and used during pupal stages for metamorphosis [9,20,21]. In both females and males, on days 0 and 1, the fat body in adults is the larval-form fat body [6]. The high expression levels of *Bdfbp1* in adults were consistent with the appearance of the larval-form fat body. The developmental transcriptional expression profiles suggest that *Bdfbp1* is mainly synthesized from the larval-form fat body for energy storage [6].

The study of *fbp1* has mainly focused on the ecdysone regulation of *fbp1* expression [7,8]. In this study, knockdown of *Bdfbp1* using RNAi was performed to understand the possible function of Bdfbp1. There are three main methods to apply double strand RNA: injection, topical application, and feeding [22]. Topical application and feeding were applied in this study since larvae were immersed in and digested the artificial diet mixed with ds*Bdfbp1* RNA. Ds*Bdfbp1* RNA was added to the artificial diet at the time before the expression of *Bdfbp1* (Figure 5a). At this time larvae were in the early third instar which were sensitive to RNAi treatment [23]. After RNAi treatment, the expression level of *Bdfbp1* was decreased without statistical difference (Figure 6b). It is possible that the concentration of ds*Bdfbp1* RNA applied is not enough. The eclosion rate was significantly decreased after RNAi treatment. The results suggest that *Bdfbp1* may be associated with the eclosion rate. Knockdown of larval storage proteins should result in a shortage of materials for adult tissue formation, and this further inhibits adult development. The larval storage proteins are mostly hexamerin [11,24,25,26]. In *Riptortus pedestris*, hexmerin-β is highly expressed in the last instar nymphs. Knockdown of hexmerin-β by RNAi in second instar nymphs delays adult emergence time [27].

Microinjection of dsRNA is useful to investigate the function of a gene. Most studies of gene functions in *B*. *dorsalis* larvae are performed in microinjection [23,28,29,30]. However, microinjection methods are not applicable for pest control. RNAi by feeding in *B*. *dorsalis* larvae is spare. Li et al. (2017) [31] fed *B*. *dorsalis* larvae ds*BdTry*s RNA on solid artificial diets, which was inconvenient for dsRNA. In this study, wet artificial diets were applied. It was easy to operate but the concentration of dsRNA would be higher to achieve the knockdown effect. Mixed ds*BdTrys* RNA treatment significantly decreased the size of *B*. *dorsalis* larvae [31]. Effect of mixed of larval storage protein dsRNA on adult development could be the future study. This should be a useful candidate for pest control based on RNAi.

## Figures and Tables

**Table 1:** Sequences of specific primers used for the rapid amplification of cDNA ends (RACE).

3′ RACE Primer	Sequence of Primer	Tm
BdAr 640-01	GGTCACCTATACTGAGGTTGTGGACGTG	60 °C
BdAr 640-03	GAACGCCAACAATCTGCAACAACTTGGC	60 °C
**5′ RACE Primer**	**Sequence of Primer**	**Tm**
BdAr 640-02	GACTTCCCTGTTGTATCCATTGAATTGGCG	58 °C
BdAr 640-06	GCTCAGTTGACTGCTGGTGTATGGTTGC	60 °C
BdArR5’-08	GCCAGCTCACGATATTGTTCGGCATTG	58 °C
BdArR5’-10	GCGGTCCATAGGGGAGTACACGATAG	60 °C
BdArR5’-12	GAGATCCCTGATTCTGTTGACGCCCATAG	58 °C
BdArR5’-14	GTCCCTGTTTCTGTTGACGCCCGTAG	60 °C

**Table 2:** Sequences of specific primers used in the real-time quantitative PCR.

Gene.	Forward Primer	Reverse Primer	Tm
fbp1	CGTGGCTCTGCTAATGGAGGTTTGC	GTCTACACCGACAGCAGCGATTCC	65 °C
gapdh	GATGACCACTGTACATGCAACCACTG	GGTCAGCTTGCCGTTCAATTCAGG	62 °C
18s	CTTAGTTCGTGGAGTGATTTGTCTG	CCAACAGGTACGACTCCACTTATATAAAC	60 °C

**Table 3:** Sequences of specific primers used for the synthesis of ds*Bdfbp1* RNA.

Primer Name	Sequence of Primer
BdArR-dsRNA-05	TAATACGACTCACTATAGGGAGATGACGTAGTGGTGATTGGTCGTG
BdArR-dsRNA-06	TAATACGACTCACTATAGGGAGATCCACGTCCACAACCTCAGTATAGG
BdArR-dsRNA-07	TAATACGACTCACTATAGGGATTGTTGTGCCGGTAGAAGG
BdArR-dsRNA-08	TAATACGACTCACTATAGGGGTTGACGCCCGTAGGAATAA

## Data Availability

Data are contained within the article.
